# The impact of alcohol consumption on the relationship between depression and chronic diarrhea: a cross-sectional study analysis on NHANES (2005-2010)

**DOI:** 10.3389/fpsyt.2024.1393546

**Published:** 2024-08-30

**Authors:** Yongsen Wang, Xiaotong Li, Zhiqun Cao, Yongkun Zhou

**Affiliations:** ^1^ First Clinical Medical College, Shandong University of Traditional Chinese Medicine, Jinan, China; ^2^ Gastroenterology Department, Affiliated Hospital of Shandong University of Traditional Chinese Medicine, Jinan, China; ^3^ Gastrointestinal and Hernia Surgery, Affiliated Hospital of Shandong University of Traditional Chinese Medicine, Jinan, China

**Keywords:** drinking, depression, diarrhea, NHANES, cross-sectional study

## Abstract

**Background:**

Alcohol consumption, depression, and chronic diarrhea are all public health issues of concern, with irreversible consequences for individual health and significant economic burdens on health care systems. Previous studies have shown that depression increases the risk of developing chronic diarrhea, but few studies have explored whether alcohol consumption has an effect on the relationship between depression and chronic diarrhea.

**Objective:**

To explore the effect of alcohol consumption on the relationship between depression and chronic diarrhea.

**Methods:**

12,538 adults (≥20 years) in NHANES from 2005-2010 were analyzed. Participants were stratified according to drinking status, and differences between the risk of depression and chronic diarrhea among participants who drank alcohol or not were assessed using multiple regression analysis and likelihood ratio tests.

**Results:**

In this cross sectional, after adding possible confounders, the prevalence of depression with chronic diarrhea was higher in the drinking population than in the non-drinking population (OR,2.34, 95%CI:1.84-2.98 and 1.26, 95%CI:0.85-1.86), with a likelihood ratio test of *P*=0.024.

**Conclusion:**

Our findings suggest that there is a significant association between depression and chronic diarrhea and that alcohol consumption may increase the correlation between depression and chronic diarrhea. However, these findings require further prospective studies to provide more evidence.

## Introduction

Depression is a type of illness caused by various factors, with low mood as the main symptom. It is characterized by loss of interest, feelings of guilt, difficulty concentrating, decreased appetite, suicidal thoughts, and other cognitive, behavioral, and social functional abnormalities (1). According to World Health Organization (WHO) statistics, approximately 350 million people worldwide are currently suffering from depression. In the United States, the 12-month and lifetime prevalence rates of depression are 10.4% and 20.6%, respectively, with a higher incidence among females than males (2–4). In both developed and developing countries, depression is the second leading cause of the global disease burden (5, 6). Depression can increase the risk of developing diabetes, heart disease, hypertension, obesity, cancer, cognitive impairments, and Alzheimer’s disease (7–13). Some studies suggest that the occurrence of chronic diarrhea is closely associated with depression (14, 15). The all-cause mortality rate for depression is around 10%. If accompanied by other comorbidities, the risk of mortality increases by 60-80%. Studies have shown that eliminating depression entirely could lead to a 10% decrease in mortality rate (1, 16, 17).

Diarrhea is defined as passing loose or liquid stools three or more times a day and/or having a stool weight exceeding 200 grams (18). The distinction between chronic diarrhea and acute diarrhea mainly depends on the duration: 4 weeks is a commonly used dividing line (19). In global statistics, the prevalence of chronic diarrhea is approximately 3-20% (20). Chronic diarrhea does not increase the risk of mortality in patients, but the detection rates of organic diseases such as colonic polyps and ulcerative colitis are significantly higher compared to other populations (21), This greatly impacts human health and quality of life, while also causing a substantial economic burden on society.

In our perception, individuals with depression often engage in alcohol abuse, and research has indeed confirmed the correlation between the two (22, 23). However, it is still unknown whether there is a change in the risk of diarrhea among individuals with depression who consume alcohol. Therefore, we are conducting a study to investigate the impact of alcohol consumption on the relationship between depression and diarrhea.

## Methods

### Study design and data source

This study was designed as a cross-sectional analysis using data from the National Health and Nutrition Examination Survey (NHANES) database (https://www.cdc.gov/nchs/nhanes/index.htm), which is collected by the National Center for Health Statistics (NCHS). NHANES gathers valuable health statistics that are utilized by public health officials, legislators, and doctors to formulate effective health policies, develop health programs and services, and enhance knowledge about national health concerns. The survey findings will aid in determining the prevalence of major diseases and risk factors associated with them. Additionally, the information will be used to assess nutritional status and its correlation with health promotion and disease prevention. Data in NHANES were de-identified and the study was approved by the National Ethical Review Board for Research in Health Statistics (ERB) (https://www.cdc.gov/nchs/nhanes/irba98.htm). This study was conducted in accordance with relevant guidelines and regulations (https://www.cdc.gov/nchs/data_access/restrictions.htm).

### Study population

**Figure 1:**
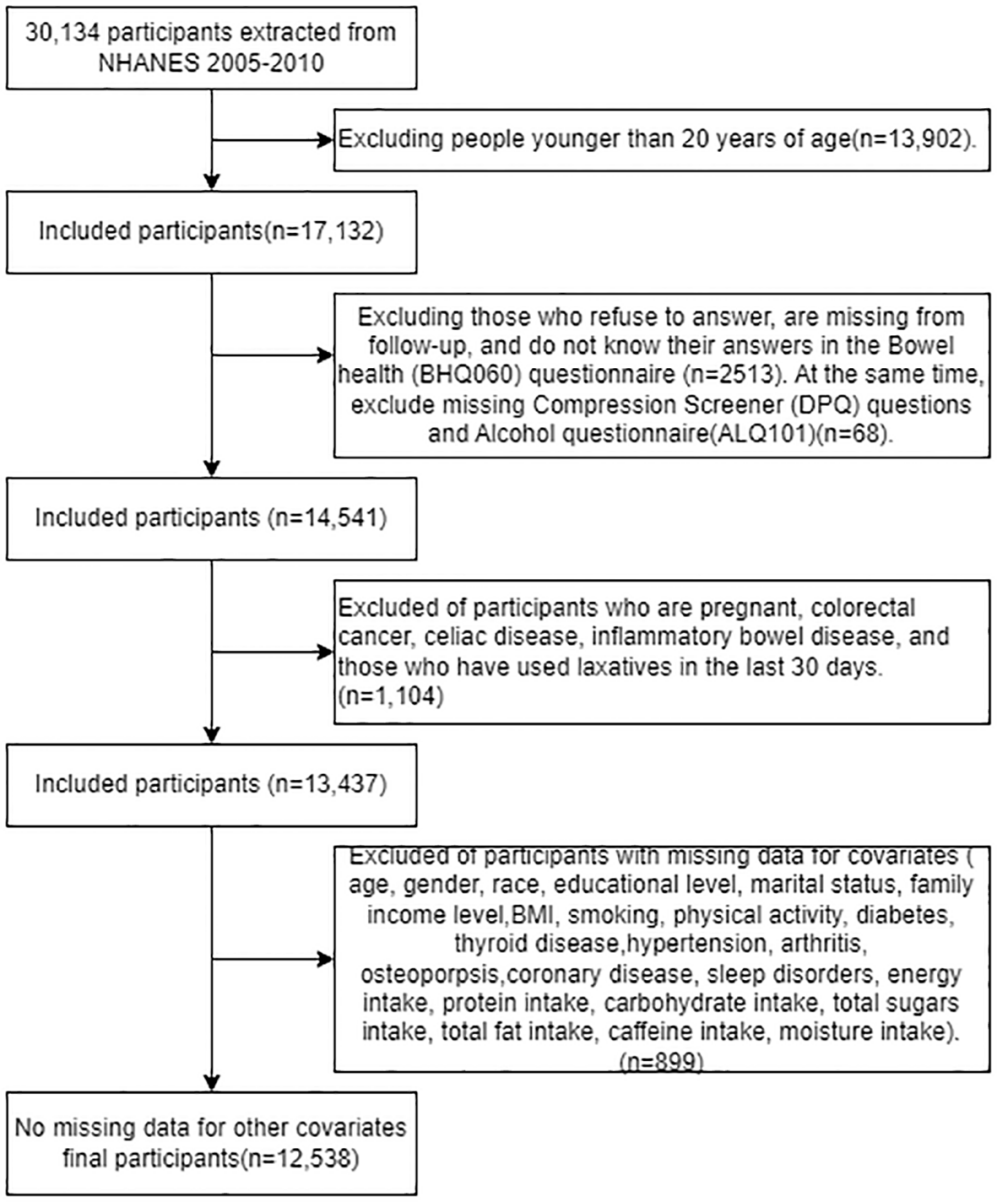
Participant selection flow chart.

Participants were drawn from NHANES records from mid-2005-2010, as these were the only three cycles in which the bowel health portion of the Mobile Examination Center (MEC) interview provided personal interview data on fecal incontinence and bowel function in adults aged 20 years and older (24). We extracted data from 20,134 participants in the NHANES dataset from 2005 to 2010; among them, 17,132 adults (≥20 years old) completed the gastrointestinal health questionnaire. Excluding invalid information from the gastrointestinal health questionnaire, depression screening questionnaire, and alcohol use questionnaire (n=2581), as well as excluding pregnant individuals, patients with colorectal cancer, individuals with abdominal diseases, those suffering from inflammatory bowel disease, and those who had used laxatives in the past 30 days (n=1104). After excluding participants with missing covariate data (n=899), 12,538 participants were included in our analysis, the exclusion criteria flowchart is shown in **Figure 1**.

### Alcohol use question and definition of drinkers

The Alcohol Use Questionnaire (ALQ) was administered in the MEC using a computer-assisted personal interview system only to medical examiners aged 20 years and older. On the questionnaire, those who consumed at least 12 alcoholic beverages in a year and answered “yes” were identified as drinkers. Among them, the number of people defined as drinking alcohol is 9102, and the number of people who do non-drinking is 3436.

### Depression screener and definition of depression

Depression is measured by the Patient Health Questionnaire (PHQ), a self-reported assessment based on nine signs and symptoms of depression based on The Diagnostic and Statistical Manual of Mental Disorders (DSM-IV), and is a tool for the initial diagnosis of depression, referred to in NHANES as the Depression Screening Questionnaire (DPQ). The nine symptom questions on the DPQ are scored on a scale from “0” (not at all) to “3” (almost every day.) The PHQ is most commonly used in primary care settings and has been shown to be a reliable and valid tool for the diagnosis of depression (25, 26). A score of 10 or greater has been well validated and is commonly used to define depression in clinical studies (25, 26).

### Bowel health questionnaire and definition of chronic diarrhea

The Bowel Health Questionnaire was completed in the MEC using a computer-assisted personal interview system. Subjects were presented with a card with a colorful picture and a description of the Bristol Stool Form Scale (BSFS) types (Type 1 to Type 7) and were asked to answer the “number corresponding to the usual or most common type of stool”. Subjects were classified as having chronic diarrhea only if they thought their usual or most common stool type was Bristol Stool Form Scale type 6 (fluffy edges, pasty) or type 7 (watery, no solid pieces) (14, 27).

### Data collection

Data collection based on demographics characteristics(age, gender, race, marital status, education level, family income); lifestyle characteristics (smoking, sleep disorder, physical activity); dietary status (protein intake, energy intake, carbohydrate intake, dietary fiber intake, total fat intake, caffeine intake, moisture intake) and comorbidity(hypertension, diabetes, arthritis, coronary disease, thyroid disease, osteoporosis).

The demographic characteristics contained age, gender (male or female), race (Mexican American, Other Hispanic, Non-Hispanic White, Non-Hispanic Black, Other Race), marital status (married, widowed/divorced and never married), education level (<12th grad and high school or above) and family income (< 20,000$ or ≥ 20,000$).

The lifestyle characteristics included smoking, physical activity and sleep disorders. Smokers are defined as those who smoke more than 100 cigarettes in their lifetime. Physical activities is divided into vigorous activities and moderate activities. The vigorous activities were defined by doing any vigorous activities for at least 10 minutes that caused heavy sweating, or large increases in breathing or heart rate over the past 30 days. The moderate physical activity was defined by doing moderate activities for at least 10 minutes that cause only light sweating or a slight to moderate increase in breathing or heart rate over the past 30 days. Participants who are informed by doctors or other health professionals of sleep disorders are defined as having sleep disorders.

Energy intake, protein intake, carbohydrate intake, total sugar intake, dietary fiber intake, total fat intake, caffeine intake, and moisture intake were collected during the first 24 hours prior to the interview through dietary recall interviews conducted at the Dietary Survey Center.

Definitions of hypertension, diabetes, arthritis, coronary heart disease, thyroid disease, and osteoporosis were taken from the Medical Condition Questionnaire, in which a doctor or other healthcare professional asked: Have you ever been told by a doctor or healthcare professional that you have a related condition? Respondents who answered “yes” were considered to have the disease.

### Statistical analysis

Data are expressed as mean± standard deviation(SD) for continuous variable and as frequency or percentage for categorical variables. The t-test was used to analyze the normal distribution and the Kruskal-Wallis test to analyze the skewed distribution in continuous variables. We used univariate and multivariate binary logistic regression to analyze the association between depression and chronic diarrhea. In multivariate logistic regression, we showed unadjusted models. Model I adjusted gender, age, race, education level, marital status, family income; Model II adjusted gender, age, race, education level, marital status, family income, smoking, BMI, high intensity exercise, moderate intensity exercise, sleep disorders, hypertension, diabetes, arthritis, coronary disease, thyroid disease, osteoporosis; Model III adjusted gender, age, race, education level, marital status, family income, smoking, BMI, high intensity exercise, moderate intensity exercise, sleep disorders, hypertension, diabetes, arthritis, coronary disease, thyroid disease, osteoporosis, energy intake, protein intake, carbohydrate intake, total sugars intake, dietary fiber intake, total fat intake, caffeine intake, moisture intake. We compared the risk of depression and chronic diarrhea between people who drank alcohol and those who did not, multivariate logistic regression analyzed subgroups according to drinking status, examined interactions between subgroups by likelihood ratios, and demonstrated trends between subgroups using smoothed curve fitting. Finally, we performed multivariate logistic regression for depression and chronic diarrhea separately using alcohol consumption as the dependent variable to verify the stability of the results. All analyses were performed using the statistical package R 4.3.2 (http://www.R-project.org), and *P* < 0.05 was considered statistically significant.

## Results

### Baseline characteristics of the study population

**Table 1** presents a comparison of baseline demographic and clinical characteristics between the drinking group and the non-drinking group. Compared to the non-drinking group, drinking group was more common in males, younger individuals, non-Hispanic white individuals, those with higher education and income, married individuals, and mostly smokers. Additionally, the alcohol-consuming group had higher intakes of alcohol, protein, sugar, fiber, fat, caffeine, and water. Furthermore, we also included baseline demographic information for individuals with and without depression, as well as those with and without diarrhea, in **Supplementary Tables 1** and **2**.

**Table 1:** Baseline characteristics of participants.

Variables	Total (n = 12538)	drinking		p
		No(n = 3436)	Yes (n = 9102)	
gender, n (%)				< 0.001
Female	6157 (49.1)	2390 (69.6)	3767 (41.4)	
Male	6381 (50.9)	1046 (30.4)	5335 (58.6)	
age (years), Mean ± SD	49.0 ± 17.6	52.2 ± 18.1	47.8 ± 17.3	< 0.001
race, n (%)				< 0.001
Mexican American	2230 (17.8)	658 (19.2)	1572 (17.3)	
Other Hispanic	1010 (8.1)	324 (9.4)	686 (7.5)	
Non-Hispanic White	6273 (50.0)	1376 (40)	4897 (53.8)	
Non-Hispanic Black	2527 (20.2)	891 (25.9)	1636 (18)	
Other Race	498 (4.0)	187 (5.4)	311 (3.4)	
education, n (%)				< 0.001
<12th Grad	6422 (51.2)	1988 (57.9)	4434 (48.7)	
High school or above	6116 (48.8)	1448 (42.1)	4668 (51.3)	
Marital status, n (%)				< 0.001
Married	6707 (53.5)	1842 (53.6)	4865 (53.4)	
Widowed/Divorced	2326 (18.6)	764 (22.2)	1562 (17.2)	
Never married	3505 (28.0)	830 (24.2)	2675 (29.4)	
Family income, n (%)				< 0.001
<20,000	3002 (24.1)	958 (28.1)	2044 (22.6)	
≥20,000	9464 (75.9)	2456 (71.9)	7008 (77.4)	
Diarrhea, n (%)				< 0.001
No	11598 (92.5)	3126 (91)	8472 (93.1)	
Yes	940 (7.5)	310 (9)	630 (6.9)	
BMI, Mean ± SD	29.0 ± 6.8	30.0 ± 7.6	28.7 ± 6.4	< 0.001
hypertension, n (%)				< 0.001
No	8278 (66.0)	2055 (59.8)	6223 (68.4)	
Yes	4260 (34.0)	1381 (40.2)	2879 (31.6)	
diabetes, n (%)				< 0.001
No	10915 (87.1)	2821 (82.1)	8094 (88.9)	
Yes	1397 (11.1)	550 (16)	847 (9.3)	
boundary	226 (1.8)	65 (1.9)	161 (1.8)	
Depression score, Mean ± SD	3.1 ± 4.1	3.1 ± 4.2	3.0 ± 4.1	0.33
Depression, n (%)				0.265
No	11510 (91.8)	3139 (91.4)	8371 (92)	
Yes	1028 (8.2)	297 (8.6)	731 (8)	
Protein intake gm, Mean ± SD	81.5 ± 43.2	70.7 ± 36.7	85.6 ± 44.7	< 0.001
arthritis, n (%)				< 0.001
No	9185 (73.3)	2354 (68.5)	6831 (75)	
Yes	3353 (26.7)	1082 (31.5)	2271 (25)	
Coronary disease, n (%)				0.362
No	12059 (96.2)	3296 (95.9)	8763 (96.3)	
Yes	479 (3.8)	140 (4.1)	339 (3.7)	
Thyroid disease, n (%)				< 0.001
No	11364 (90.6)	2988 (87)	8376 (92)	
Yes	1174 (9.4)	448 (13)	726 (8)	
osteoporosis, n (%)				< 0.001
No	11865 (94.6)	3146 (91.6)	8719 (95.8)	
Yes	673 (5.4)	290 (8.4)	383 (4.2)	
High intensity exercise, n (%)				< 0.001
No	9534 (76.0)	2828 (82.3)	6706 (73.7)	
Yes	3004 (24.0)	608 (17.7)	2396 (26.3)	
Moderate intensity exercise, n (%)				< 0.001
No	7086 (56.5)	2105 (61.3)	4981 (54.7)	
Yes	5452 (43.5)	1331 (38.7)	4121 (45.3)	
smoking, n (%)				< 0.001
No	6546 (52.2)	2455 (71.4)	4091 (44.9)	
Yes	5992 (47.8)	981 (28.6)	5011 (55.1)	
Sleep disorders, n (%)				0.456
No	11621 (92.7)	3175 (92.4)	8446 (92.8)	
Yes	917 (7.3)	261 (7.6)	656 (7.2)	
Energy intake kcal (Mean ± SD)	2126.9 ± 1018.3	1829.9 ± 858.7	2239.0 ± 1050.8	< 0.001
Carbohydrate intake gm (Mean ± SD)	257.8 ± 128.1	236.1 ± 115.8	266.0 ± 131.5	< 0.001
Total sugars intake gm (Mean ± SD)	117.5 ± 80.4	110.4 ± 72.8	120.2 ± 82.9	< 0.001
Dietary fiber intake gm (Mean ± SD)	16.0 ± 9.8	15.2 ± 9.2	16.3 ± 10.1	< 0.001
Total fat intake gm (Mean ± SD)	79.7 ± 46.8	69.3 ± 40.6	83.7 ± 48.4	< 0.001
Caffeine intake mg (Mean ± SD)	102.0 (17.0, 225.8)	72.0 (6.0, 161.0)	120.0 (29.0, 252.0)	< 0.001
Moisture intake gm (Mean ± SD)	2900.9 ± 1489.8	2450.4 ± 1234.0	3071.0 ± 1541.9	< 0.001

### Depression affects the risk of developing diarrhea

The univariate analysis in **Table 2** shows that gender, age, race, education level, marital status, income level, BMI, hypertension, diabetes, arthritis, coronary heart disease, thyroid disorders, osteoporosis, physical activity level, smoking, sleep disorders, and dietary fiber intake are associated with chronic diarrhea.

**Table 2:** The relationship between covariates and the risk of chronic diarrhea.

Variable	OR_95CI	P_value
gender, n (%)		
Male	0.72 (0.63~0.82)	<0.001
Female	1 (reference)	
Age	1.01 (1.01~1.02)	<0.001
race, n (%)		
Mexican American	1 (reference)	
Other Hispanic	0.91 (0.7~1.19)	0.509
Non-Hispanic White	0.72 (0.6~0.86)	<0.001
Non-Hispanic Black	0.92 (0.75~1.12)	0.394
Other Race	0.82 (0.57~1.18)	0.284
education, n (%)		
<12th Grad	1 (reference)	
High School Grad/GED	0.61 (0.53~0.7)	<0.001
Marital status, n (%)		
Married	1 (reference)	
Widowed/Divorced	1.15 (0.97~1.36)	0.113
Never married	0.81 (0.69~0.95)	0.01
Family income, n (%)		
<20,000	1 (reference)	
≥20,000	0.67 (0.58~0.77)	<0.001
BMI	1.03 (1.02~1.04)	<0.001
hypertension, n (%)		
No	1 (reference)	
Yes	1.66 (1.45~1.9)	<0.001
diabetes, n (%)		
No	1 (reference)	
Yes	1.91 (1.6~2.27)	<0.001
boundary	2.1 (1.42~3.11)	<0.001
arthritis, n (%)		
No	1 (reference)	
Yes	1.51 (1.31~1.74)	<0.001
Coronary disease, n (%)		
No	1 (reference)	
Yes	1.13 (0.81~1.57)	0.47
Thyroid disease, n (%)		
No	1 (reference)	
Yes	1.3 (1.05~1.6)	0.015
osteoporosis, n (%)		
No	1 (reference)	
Yes	1.52 (1.18~1.96)	0.001
High intensity exercise, n (%)		
No	1 (reference)	
Yes	0.7 (0.59~0.83)	<0.001
Moderate intensity exercise, n (%)		
No	1 (reference)	
Yes	0.74 (0.64~0.85)	<0.001
smoking, n (%)		
No	1 (reference)	
Yes	1.23 (1.08~1.4)	0.002
Sleep disorders, n (%)		
No	1 (reference)	
Yes	1.51 (1.21~1.89)	<0.001
Energy intake kcal	1 (1~1)	<0.001
Protein intake gm	1 (1~1)	0.001
Carbohydrate intake gm	1 (1~1)	<0.001
Total sugars intake gm	1 (1~1)	0.001
Dietary fiber intake gm	0.99 (0.98~1)	0.021
Total fat intake gm	1 (1~1)	<0.001
Caffeine intake mg	1 (1~1)	0.934
Moisture intake gm	1 (1~1)	0.192

**Table 3** presents the results of the logistic regression analysis. In the unadjusted model, the risk of developing diarrhea in the depressed population is 2.36 times that of the non-depressed population (95% CI, 1.96-2.85); after adjusting for baseline variables (gender, age, race, education level, marital status, household income), the results remain similar (OR, 2.18; 95% CI, 1.79-2.64). After adjusting for other potential confounding factors (BMI, high intensity exercise, moderate intensity exercise, sleep disorders, hypertension, diabetes, arthritis, coronary disease, thyroid disease, osteoporosis, energy intake, protein intake, carbohydrate intake, total sugars intake, dietary fiber intake, total fat intake, caffeine intake, moisture intake) using a multivariable regression model, the risk of diarrhea in the depressed population was 1.94 times that of the non-depressed population (95% CI, 1.58-2.37). When depression was transformed into different levels of severity based on the scores, a significant relationship was observed in different adjusted models (*P* < 0.05). Additionally, we analyzed depression scores as a continuous variable, and the results remained stable.

**Table 3:** Weighted odds ratios (95% confidence interval) for chronic diarrhea and different levels of depression in different models.

Variable	total	event (%)	Non-adjusted model		Model I		Model II		Model III	
			OR (95%CI)	p value	OR (95%CI)	p value	OR (95%CI)	p value	OR (95%CI)	p value
Non-depressed participants	11510	788 (6.8%)	1(Ref)		1(Ref)		1(Ref)		1(Ref)	
Depressed participants	1028	152 (14.8%)	2.36 (1.96~2.85)	<0.001	2.18 (1.79~2.64)	<0.001	1.94 (1.59~2.37)	<0.001	1.94 (1.58~2.37)	<0.001
Subgroups										
≤9	11294	762 (6.7%)	1(Ref)		1(Ref)		1(Ref)		1(Ref)	
10-15	878	114 (13%)	2.06 (1.67~2.54)	<0.001	1.95 (1.57~2.42)	<0.001	1.78 (1.42~2.22)	<0.001	1.77 (1.42~2.21)	<0.001
16-21	290	49 (16.9%)	2.81 (2.05~3.85)	<0.001	2.54 (1.84~3.51)	<0.001	2.29 (1.65~3.19)	<0.001	2.32 (1.67~3.24)	<0.001
≥22	76	15 (19.7%)	3.4 (1.92~6.01)	<0.001	3 (1.68~5.34)	<0.001	2.49 (1.38~4.48)	0.002	2.43 (1.35~4.39)	0.003
*P*-Trend			1.69 (1.52~1.88)	<0.001	1.61 (1.44~1.8)	<0.001	1.51 (1.34~1.69)	<0.001	1.51 (1.34~1.69)	<0.001
Depression score	12538	940(7.5%)	1.07 (1.06-1.09)	<0.001	1.07 (1.05-1.08)	<0.001	1.06 (1.05-1.08)	<0.001	1.06 (1.04-1.07)	<0.001

Model I: adjusted gender, age, race, education level, marital status, family income.

Model II: adjusted gender, age, race, education level, marital status, family income, smoking, BMI, high intensity exercise, moderate intensity exercise, sleep disorders, hypertension, diabetes, arthritis, coronary disease, thyroid disease, osteoporosis.

Model III: adjusted gender, age, race, education level, marital status, family income, smoking, BMI, high intensity exercise, moderate intensity exercise, sleep disorders, hypertension, diabetes, arthritis, coronary disease, thyroid disease, osteoporosis, energy intake, protein intake, carbohydrate intake, total sugars intake, dietary fiber intake, total fat intake, caffeine intake, moisture intake.

### The association between depression and the risk of diarrhea in populations based on drinking

**Figure 2:**
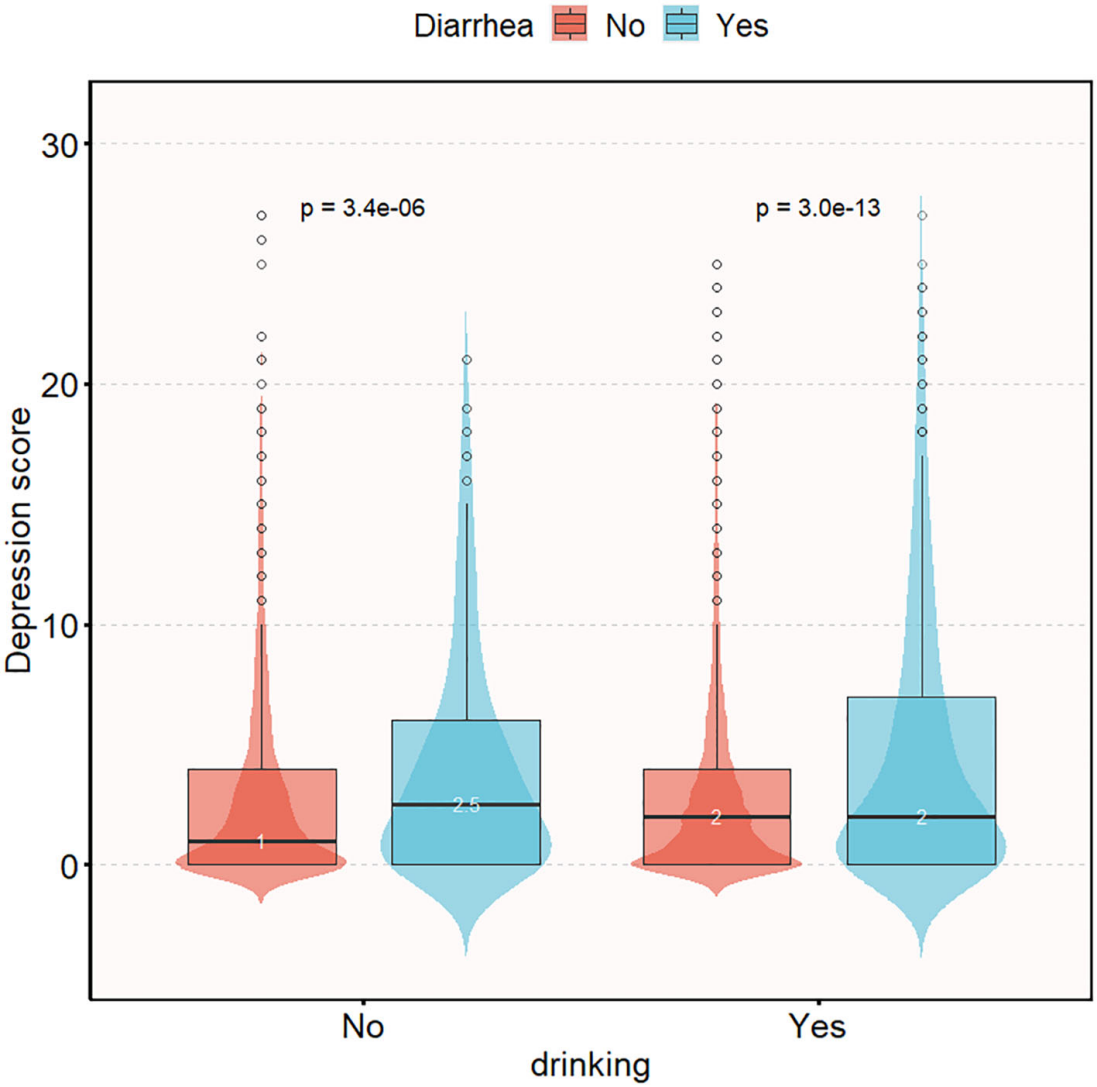
Distribution of depression questionnaire scores in chronic diarrhea population grouped according to drinking status. Adjusted gender, age, race, education level, marital status, family income, smoking, BMI, high intensity exercise, moderate intensity exercise, sleep disorders, hypertension, diabetes, arthritis, coronary disease, thyroid disease, osteoporosis, energy intake, protein intake, carbohydrate intake, total sugars intake, dietary fiber intake, total fat intake, caffeine intake, moisture intake.

**Figure 2** illustrates the difference in depression questionnaire scores between the alcohol-consuming and non-alcohol-consuming populations. Among the alcohol-consuming group, the depression scores were similar between the diarrhea and non-diarrhea subgroups (2 vs 2, *P* < 0.05); however, in the non-alcohol-consuming group, the diarrhea subgroup had higher depression scores compared to the non-diarrhea subgroup (1 vs 2.5, *P* < 0.05). 

**Table 4** illustrates that after adjusting for multiple confounding factors (gender, age, race, education level, marital status, family income, smoking, BMI, high intensity exercise, moderate intensity exercise, sleep disorders, hypertension, diabetes, arthritis, coronary disease, thyroid disease, osteoporosis, energy intake, protein intake, carbohydrate intake, total sugars intake, dietary fiber intake, total fat intake, caffeine intake, moisture intake), the risk of chronic diarrhea in the depressed population compared to the non-depressed population is 2.34 in the alcohol-consuming group (95% CI, 1.84-2.98; *P* < 0.05). However, in the non-alcohol-consuming group, there was no significant interaction between depression and chronic diarrhea (OR: 1.26; 95% CI, 0.85-1.86; *P* > 0.05). Compared to the non-drinking population, the probability of chronic diarrhea occurring in patients with depression is 2.34 times higher in the drinking population than in non-depressed patients (95% CI: 1.84-2.98); this interaction remained significant when depression was transformed into different levels of severity based on scores (likelihood ratio test, *P* = 0.043). Finally, analyzing depression scores as a continuous variable showed that the risk of diarrhea in the depressed population was lower in the non-alcohol-consuming group compared to the alcohol-consuming group (likelihood ratio test, *P* = 0.024).

**Table 4:** Interaction between depression levels and chronic diarrhea in patients with alcohol consumption.

Variable	Non-drinking(n=3,436)		Drinking(n=9,102)		P for interaction
	OR (95%CI)	p value	OR (95%CI)	p value	
Non-depressed participants	1(Ref)		1(Ref)		0.024
Depressed participants	1.26 (0.85~1.86)	0.247	2.34 (1.84~2.98)	<0.001	
Subgroup					
≤9	1(Ref)		1(Ref)		0.043
10-15	1.24 (0.82~1.88)	0.312	2.08 (1.6~2.71)	<0.001	
16-21	1.6 (0.86~2.97)	0.136	2.82 (1.9~4.19)	<0.001	
≥22	0.6 (0.13~2.74)	0.511	3.87 (2.01~7.46)	<0.001	
P-Trend	1.13 (0.9~1.43)	0.287	1.71 (1.49~1.96)	<0.001	
Depression score	1.03 (1-1.06)	0.041	1.07 (1.06-1.09)	<0.001	0.024

Data presented are ORs and 95% CIs.

Adjusted for gender, age, race, education level, marital status, family income, smoking, BMI, high intensity exercise, moderate intensity exercise, sleep disorders, hypertension, diabetes, arthritis, coronary disease, thyroid disease, osteoporosis, energy intake, protein intake, carbohydrate intake, total sugars intake, dietary fiber intake, total fat intake, caffeine intake, moisture intake.

### The smooth fitting curve of depression scores and the risk of chronic diarrhea

**Figure 3:**
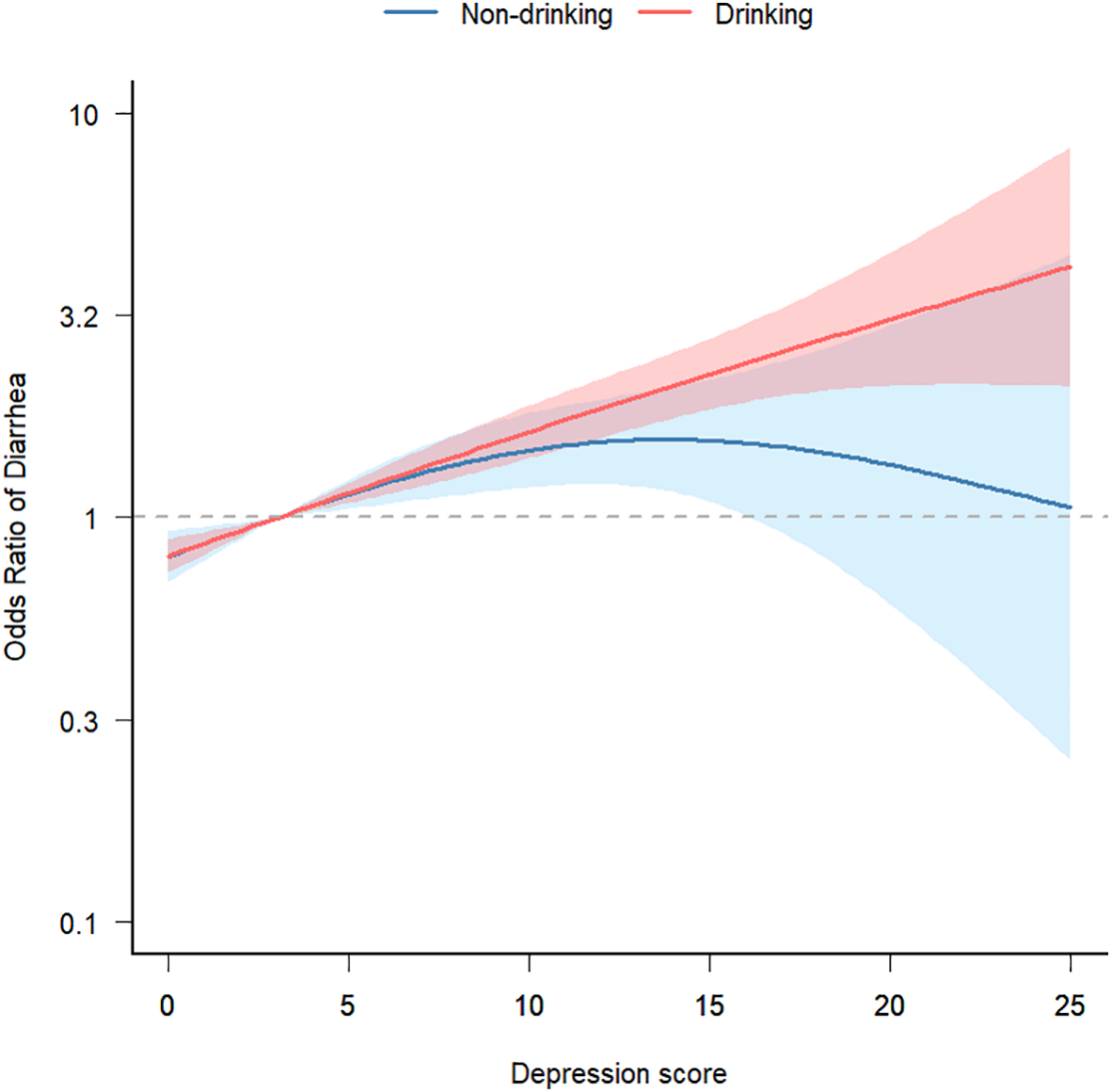
Smooth fit curve of depression score and risk of chronic diarrhea. Adjusted gender, age, race, education level, marital status, family income, smoking, BMI, high intensity exercise, moderate intensity exercise, sleep disorders, hypertension, diabetes, arthritis, coronary disease, thyroid disease, osteoporosis, energy intake, protein intake, carbohydrate intake, total sugars intake, dietary fiber intake, total fat intake, caffeine intake, moisture intake.

Finally, after adjusting for potential confounding factors (gender, age, race, education level, marital status, family income, smoking, BMI, high intensity exercise, moderate intensity exercise, sleep disorders, hypertension, diabetes, arthritis, coronary disease, thyroid disease, osteoporosis, energy intake, protein intake, carbohydrate intake, total sugars intake, dietary fiber intake, total fat intake, caffeine intake, moisture intake), we conducted a smooth fitting curve analysis of depression scores and the risk of chronic diarrhea. In **Figure 3**, we grouped individuals based on alcohol consumption and found that when depression scores were low, alcohol consumption had little effect on the relationship between depression and chronic diarrhea. However, as depression scores increased, individuals who consumed alcohol had a higher risk of developing diarrhea.

### Sensitivity analysis

To control for the independent effects of alcohol consumption on depression and chronic diarrhea, we conducted separate multiple regression analyses using alcohol consumption as a variable for depression and chronic diarrhea. The results indicated that in the unadjusted model, alcohol consumption had a protective effect against chronic diarrhea; however, this association lost significance after adjusting for confounding factors. Interestingly, in the logistic regression between alcohol consumption and depression, the unadjusted model showed a protective effect of alcohol consumption on depression, which remained non-significant. What’s intriguing is that this result flipped in models I and II, where the risk of developing depression in the alcohol-consuming group increased by 18%(P=0.038) and 25%(P=0.005) compared to the non-alcohol-consuming group. However, in model III, the significance disappeared (*P*=0.273). We believe that some of the confounding factors were strongly correlated with depression or had strong negative correlations, leading to these results. **Supplementary Tables 3** and **4** display the results of the multiple logistic regression analyses between alcohol consumption and depression, as well as chronic diarrhea.

## Discussions

Based on a cross-sectional analysis of U.S. adults (≥20 years) from NHANES(2005-2010), we found that after adjusting for potential important confounding factors, depression is positively associated with the risk of chronic diarrhea occurrence. This result is consistent with several previous studies (14, 15). The association between functional gastrointestinal disorders and mental illnesses is well known to most clinical practitioners. A cross-sectional survey conducted by Wang found a significant correlation between fecal incontinence and depression, with depression increasing the risk of fecal incontinence (28). Gorard (29), in a study involving 42 individuals in a general psychiatric outpatient clinic, measured oral-cecal transit time using lactulose hydrogen breath testing and whole gut transit time with an ingestion of a non-absorbable marker followed by abdominal X-ray imaging. The results indicated a relationship between anxiety patients and increased bowel frequency, showing that emotions can influence intestinal motility. An analysis by Mate also found a high prevalence of irritable bowel syndrome (IBS) associated with psychological disorders (30). Depression often coexists with alcohol abuse, and when we included alcohol consumption in the analysis, we found that drinking increased the risk of developing diarrhea in individuals with depression. This association remained stable even after adjusting for multiple confounding factors.

Psychological stress has significant effects on intestinal sensitivity, motility, secretion, permeability, and mucosal immune activation (31). The mechanism behind the increased risk of diarrhea in patients with depression may be related to the brain-gut axis and visceral hypersensitivity. The brain-gut axis combines the activity of the autonomic and enteric nervous systems with central nervous system regulation, with its components communicating bidirectionally, cooperatively, and antagonistically to regulate the host’s physiological equilibrium. The bidirectional regulation between the brain and gut through this axis is known as brain-gut interaction (32, 33). As an important neurotransmitter in the brain-gut axis, 5-HT can regulate gastrointestinal motility, visceral sensation, and mucosal secretion. Research has shown that elevated serum 5-HT is associated with diarrhea-predominant irritable bowel syndrome, indicating a close relationship between elevated serum 5-HT and this subtype of irritable bowel syndrome (34).

According to the Global Burden of Disease Study findings, alcohol consumption has been identified as a major factor contributing to the burden of disease and death (35). A survey (36) from China found that alcohol consumption is significantly positively correlated with depressive symptoms, similar to previous research (37, 38). After adjusting for possible confounding factors, the relationship between alcohol consumption and depression was not significant, in contrast to previous research. Meanwhile, we also analyzed the relationship between alcohol consumption and chronic diarrhea, which remained non-significant after controlling for confounders. However, our final analysis revealed that among alcohol consumers, the risk of chronic diarrhea was higher in individuals with depression.

In this study, we examined the association between depression and chronic diarrhea in both alcohol-consuming and non-alcohol-consuming populations after adjusting for relevant variables. Our study has limitations. Firstly, a cross-sectional design does not allow for causal inference. Secondly, we cannot rule out the possibility that the observed associations may be due to unmeasured confounding factors. Lastly, some variables were based on self-reporting, which could lead to issues such as misunderstanding of questions or recall bias. Despite these limitations, our study has some merits. Firstly, our study population consisted of a large, representative sample of American adults. Secondly, aside from exploring the association between depression and chronic diarrhea, we also stratified the analysis based on alcohol consumption. Lastly, we used smooth curve fitting to assess the relationship between depression and chronic diarrhea in alcohol-consuming and non-alcohol-consuming populations.

## Conclusion

In conclusion, this study found an association between depression and chronic diarrhea, with alcohol consumption increasing this association. This may alert individuals with depression to take precautions to avoid the occurrence of chronic diarrhea. Our study provides some clinical insights but further prospective research is needed to provide more evidence.

## The impact on research and practice

Our findings hold significant implications for public health policy and clinical practice. In terms of public health policy, it is crucial to increase awareness about the impact of alcohol consumption on mental and digestive health. Public awareness campaigns should be enhanced to highlight the dangers of drinking, and efforts to implement alcohol control policies should be strengthened, particularly among vulnerable populations such as individuals with depression. In clinical practice, healthcare professionals should pay close attention to the drinking habits of patients with depression and incorporate alcohol management strategies into their treatment plans. For patients exhibiting symptoms of chronic diarrhea, especially those with a history of alcohol consumption and depression, doctors should consider alcohol as a potential contributing factor and conduct comprehensive assessments and treatments accordingly.

## Data Availability

Publicly available datasets were analyzed in this study. This data can be found here: https://wwwn.cdc.gov/nchs/nhanes/Default.aspx.
