# Supplementary material for: The impact of alcohol consumption on the relationship between depression and chronic diarrhea: a cross-sectional study analysis on NHANES (2005-2010)
Source: Front Psychiatry. 2024 Aug 30;15:1393546. doi: 10.3389/fpsyt.2024.1393546 (PMC11392863; doi:10.3389/fpsyt.2024.1393546)
Supplement: Supplementary file 1 [file Table1.docx]

Supplementary Table 1 Baseline population characteristics (categorized as depressed and non-depressed populations)

| Variables | Total (n = 12538) | Diarrhea | | p |
| --- | --- | --- | --- | --- |
|  |  | No(n = 11598) | Yes (n = 940) |  |
| gender, n (%) | |  |  | < 0.001 |
| Female | 6157(49.1) | 5623(48.5) | 534(56.8) |  |
| Male | 6381(50.9) | 5975(51.5) | 406(43.2) |  |
| age (years), Mean ± SD | 49.0±17.6 | 48.7±17.7 | 53.0±16.3 | < 0.001 |
| race, n (%) | |  |  | < 0.002 |
| Mexican American | 2230(17.8) | 2031(17.5) | 199(21.2) |  |
| Other Hispanic | 1010(8.1) | 927(8) | 83(8.8) |  |
| Non-Hispanic White | 6273 (50.0) | 5860 (50.5) | 413 (43.9) |  |
| Non-Hispanic Black | 2527 (20.2) | 2319 (20) | 208 (22.1) |  |
| Other Race | 498 ( 4.0) | 461 (4) | 37 (3.9) |  |
| education, n (%) | |  |  | < 0.001 |
| <12th Grad | 6422 (51.2) | 5835 (50.3) | 587 (62.4) |  |
| High school or above | 6116 (48.8) | 5763 (49.7) | 353 (37.6) |  |
| Marital status, n (%) | |  |  | < 0.002 |
| Married | 6707 (53.5) | 6191 (53.4) | 516 (54.9) |  |
| Widowed/Divorced | 2326 (18.6) | 2123 (18.3) | 203 (21.6) |  |
| Never married | 3505 (28.0) | 3284 (28.3) | 221 (23.5) |  |
| Family income, n (%) | |  |  | < 0.001 |
| <20,000 | 3002 (24.1) | 2707 (23.5) | 295 (31.4) |  |
| ≥20,000 | 9464 (75.9) | 8820 (76.5) | 644 (68.6) |  |
| drinking, n (%) | |  |  | < 0.001 |
| No | 3436 (27.4) | 3126 (27) | 310 (33) |  |
| Yes | 9102 (72.6) | 8472 (73) | 630 (67) |  |
| BMI, Mean ± SD | 29.0 ± 6.8 | 28.9 ± 6.6 | 30.7 ± 8.2 | < 0.001 |
| hypertension, n (%) | |  |  | < 0.001 |
| No | 8278 (66.0) | 7706 (67) | 572 (55.6) |  |
| Yes | 4260 (34.0) | 3804 (33) | 456 (44.4) |  |
| diabetes, n (%) | |  |  | < 0.001 |
| No | 10915 (87.1) | 10085 (87.6) | 830 (80.7) |  |
| Yes | 1397 (11.1) | 1228 (10.7) | 169 (16.4) |  |
| boundary | 226 ( 1.8) | 197 (1.7) | 29 (2.8) |  |
| Drinking, n (%) | |  |  | 0.265 |
| No | 3436 (27.4) | 3139 (27.3) | 297 (28.9) |  |
| Yes | 9102 (72.6) | 8371 (72.7) | 731 (71.1) |  |
| Depression score, Mean ± SD | 3.1 ± 4.1 | 2.9 ± 4.0 | 4.5 ± 5.3 | < 0.001 |
| Depression, n (%) |  |  |  | < 0.001 |
| No | 11510 (91.8) | 10722 (92.4) | 788 (83.8) |  |
| Yes | 1028 ( 8.2) | 876 (7.6) | 152 (16.2) |  |
| Protein intake gm, Mean ± SD | 81.5 ± 43.2 | 81.9 ± 43.3 | 77.0 ± 41.7 | < 0.001 |
| arthritis, n (%) | |  |  | < 0.001 |
| No | 9185 (73.3) | 8572 (73.9) | 613 (65.2) |  |
| Yes | 3353 (26.7) | 3026 (26.1) | 327 (34.8) |  |
| Coronary disease, n (%) | | |  | < 0.47 |
| No | 12059 (96.2) | 11159 (96.2) | 900 (95.7) |  |
| Yes | 479 ( 3.8) | 439 (3.8) | 40 (4.3) |  |
| Thyroid disease, n (%) | | |  | < 0.015 |
| No | 11364 (90.6) | 10533 (90.8) | 831 (88.4) |  |
| Yes | 1174 ( 9.4) | 1065 (9.2) | 109 (11.6) |  |
| osteoporosis, n (%) | |  |  | < 0.001 |
| No | 11865 (94.6) | 10997 (94.8) | 868 (92.3) |  |
| Yes | 673 ( 5.4) | 601 (5.2) | 72 (7.7) |  |
| High intensity exercise, n (%) | | |  | < 0.001 |
| No | 9534 (76.0) | 8768 (75.6) | 766 (81.5) |  |
| Yes | 3004 (24.0) | 2830 (24.4) | 174 (18.5) |  |
| Moderate intensity exercise, n (%) | | |  | < 0.001 |
| No | 7086 (56.5) | 6491 (56) | 595 (63.3) |  |
| Yes | 5452 (43.5) | 5107 (44) | 345 (36.7) |  |
| smoking, n (%) | |  |  | < 0.002 |
| No | 6546 (52.2) | 6100 (52.6) | 446 (47.4) |  |
| Yes | 5992 (47.8) | 5498 (47.4) | 494 (52.6) |  |
| Sleep disorders, n (%) | | |  | 0.001 |
| No | 11621 (92.7) | 10778 (92.9) | 843 (89.7) |  |
| Yes | 917 ( 7.3) | 820 (7.1) | 97 (10.3) |  |
| Energy intake kcal, Mean ± SD | 2126.9 ± 1018.3 | 2136.9 ± 1023.8 | 2004.5 ± 939.2 | < 0.001 |
| Carbohydrate intake gm., Mean ± SD | 257.8 ± 128.1 | 258.9 ± 129.0 | 243.8 ± 115.4 | < 0.001 |
| Total sugars intake gm, Mean ± SD | 117.5 ± 80.4 | 118.1 ± 80.9 | 109.4 ± 73.6 | 0.001 |
| Dietary fiber intake gm, Mean ± SD | 16.0 ± 9.8 | 16.1 ± 9.8 | 15.3 ± 10.4 | 0.021 |
| Total fat intake gm, Mean ± SD | 79.7 ± 46.8 | 80.1 ± 47.0 | 74.4 ± 45.2 | < 0.001 |
| Caffeine intake mg, Mean ± SD | 102.0 (17.0, 225.8) | 102.0 (16.0, 226.0) | 100.0 (24.5, 216.5) | 0.87 |
| Moisture intake gm, Mean ± SD | 2900.9 ± 1489.8 | 2905.8 ± 1492.6 | 2840.0 ± 1454.3 | 0.192 |

Supplementary Table 2 Baseline Characteristics of the Population (disaggregated by diarrhea and non-diarrhea populations)

| Variables | Total (n = 12538) | Depression | | p |
| --- | --- | --- | --- | --- |
|  |  | No(n = 11510) | Yes (n = 1028) |  |
| gender, n (%) | |  |  | < 0.001 |
| Female | 6157 (49.1) | 5513 (47.9) | 644 (62.6) |  |
| Male | 6381 (50.9) | 5997 (52.1) | 384 (37.4) |  |
| age (years), Mean ± SD | 49.0 ± 17.6 | 49.2 ± 17.8 | 47.0 ± 15.3 | < 0.001 |
| race, n (%) | |  |  | < 0.001 |
| Mexican American | 2230 (17.8) | 2047 (17.8) | 183 (17.8) |  |
| Other Hispanic | 1010 ( 8.1) | 902 (7.8) | 108 (10.5) |  |
| Non-Hispanic White | 6273 (50.0) | 5811 (50.5) | 462 (44.9) |  |
| Non-Hispanic Black | 2527 (20.2) | 2291 (19.9) | 236 (23) |  |
| Other Race | 498 ( 4.0) | 459 (4) | 39 (3.8) |  |
| education, n (%) | |  |  | < 0.001 |
| <12th Grad | 6422 (51.2) | 5756 (50) | 666 (64.8) |  |
| High school or above | 6116 (48.8) | 5754 (50) | 362 (35.2) |  |
| Marital status, n (%) | |  |  | < 0.001 |
| Married | 6707 (53.5) | 6326 (55) | 381 (37.1) |  |
| Widowed/Divorced | 2326 (18.6) | 2070 (18) | 256 (24.9) |  |
| Never married | 3505 (28.0) | 3114 (27.1) | 391 (38) |  |
| Family income, n (%) | |  |  | < 0.001 |
| <20,000 | 3002 (24.1) | 2553 (22.3) | 449 (44.1) |  |
| ≥20,000 | 9464 (75.9) | 8894 (77.7) | 570 (55.9) |  |
| Diarrhea, n (%) | |  |  | < 0.001 |
| No | 11598 (92.5) | 10722 (93.2) | 876 (85.2) |  |
| Yes | 940 ( 7.5) | 788 (6.8) | 152 (14.8) |  |
| BMI, Mean ± SD | 29.0 ± 6.8 | 28.9 ± 6.6 | 30.6 ± 8.3 | < 0.001 |
| hypertension, n (%) | |  |  | < 0.001 |
| No | 8278 (66.0) | 7706 (67) | 572 (55.6) |  |
| Yes | 4260 (34.0) | 3804 (33) | 456 (44.4) |  |
| diabetes, n (%) | |  |  | < 0.001 |
| No | 10915 (87.1) | 10085 (87.6) | 830 (80.7) |  |
| Yes | 1397 (11.1) | 1228 (10.7) | 169 (16.4) |  |
| boundary | 226 ( 1.8) | 197 (1.7) | 29 (2.8) |  |
| Drinking, n (%) | |  |  | 0.265 |
| No | 3436 (27.4) | 3139 (27.3) | 297 (28.9) |  |
| Yes | 9102 (72.6) | 8371 (72.7) | 731 (71.1) |  |
| Protein intake gm, Mean ± SD | 81.5 ± 43.2 | 82.2 ± 43.0 | 73.6 ± 44.6 | < 0.001 |
| arthritis, n (%) | |  |  | < 0.001 |
| No | 9185 (73.3) | 8595 (74.7) | 590 (57.4) |  |
| Yes | 3353 (26.7) | 2915 (25.3) | 438 (42.6) |  |
| Coronary disease, n (%) | | |  | < 0.001 |
| No | 12059 (96.2) | 11090 (96.4) | 969 (94.3) |  |
| Yes | 479 ( 3.8) | 420 (3.6) | 59 (5.7) |  |
| Thyroid disease, n (%) | | |  | < 0.001 |
| No | 11364 (90.6) | 10485 (91.1) | 879 (85.5) |  |
| Yes | 1174 ( 9.4) | 1025 (8.9) | 149 (14.5) |  |
| osteoporosis, n (%) | |  |  | < 0.003 |
| No | 11865 (94.6) | 10913 (94.8) | 952 (92.6) |  |
| Yes | 673 ( 5.4) | 597 (5.2) | 76 (7.4) |  |
| High intensity exercise, n (%) | | |  | < 0.001 |
| No | 9534 (76.0) | 8691 (75.5) | 843 (82) |  |
| Yes | 3004 (24.0) | 2819 (24.5) | 185 (18) |  |
| Moderate intensity exercise, n (%) | | |  | < 0.001 |
| No | 7086 (56.5) | 6402 (55.6) | 684 (66.5) |  |
| Yes | 5452 (43.5) | 5108 (44.4) | 344 (33.5) |  |
| smoking, n (%) | |  |  | < 0.001 |
| No | 6546 (52.2) | 6141 (53.4) | 405 (39.4) |  |
| Yes | 5992 (47.8) | 5369 (46.6) | 623 (60.6) |  |
| Sleep disorders, n (%) | | |  | 0.001 |
| No | 11621 (92.7) | 10809 (93.9) | 812 (79) |  |
| Yes | 917 ( 7.3) | 701 (6.1) | 216 (21) |  |
| Energy intake kcal, Mean ± SD | 2126.9 ± 1018.3 | 2135.4 ± 1017.2 | 2032.5 ± 1026.2 | 0.002 |
| Carbohydrate intake gm., Mean ± SD | 257.8 ± 128.1 | 258.2 ± 127.7 | 253.1 ± 132.1 | 0.222 |
| Total sugars intake gm, Mean ± SD | 117.5 ± 80.4 | 117.0 ± 79.7 | 123.2 ± 87.3 | 0.018 |
| Dietary fiber intake gm, Mean ± SD | 16.0 ± 9.8 | 16.2 ± 9.9 | 13.9 ± 9.4 | < 0.001 |
| Total fat intake gm, Mean ± SD | 79.7 ± 46.8 | 80.1 ± 46.9 | 75.3 ± 46.0 | 0.002 |
| Caffeine intake mg, Mean ± SD | 102.0 (17.0, 225.8) | 102.0 (17.0, 224.0) | 108.0 (20.0, 244.2) | 0.102 |
| Moisture intake gm, Mean ± SD | 2900.9 ± 1489.8 | 2900.8 ± 1469.2 | 2902.7 ± 1705.1 | 0.969 |

Attachment Table 3 Logistic regression analysis between alcohol consumption and chronic diarrhea

| Variable | total | event (%) | Non-adjusted model | | Model Ⅰ | | Model Ⅱ | | Model Ⅲ | |
| --- | --- | --- | --- | --- | --- | --- | --- | --- | --- | --- |
|  |  |  | OR (95%CI) | p value | OR (95%CI) | p value | OR (95%CI) | p value | OR (95%CI) | p value |
| Non-drinking | 3436 | 310 (9%) | 1(Ref) | | 1(Ref) | | 1(Ref) | | 1(Ref) |  |
| Drinking | 9102 | 630 (6.9%) | 0.75 (0.65~0.86) | <0.001 | 0.95 (0.82~1.11) | 0.533 | 0.97 (0.83~1.13) | 0.654 | 0.9 (0.76~1.05) | 0.186 |

Model Ⅰ: adjusted gender, age, race, education level, marital status, family income.

Model Ⅱ: adjusted gender, age, race, education level, marital status, family income, smoking, BMI, high intensity exercise, moderate intensity exercise, sleep disorders, hypertension, diabetes, arthritis, coronary disease, thyroid disease, osteoporosis.

Model Ⅲ: adjusted gender, age, race, education level, marital status, family income, smoking, BMI, high intensity exercise, moderate intensity exercise, sleep disorders, hypertension, diabetes, arthritis, coronary disease, thyroid disease, osteoporosis, energy intake, protein intake, carbohydrate intake, total sugars intake, dietary fiber intake, total fat intake, caffeine intake, moisture intake.

Attachment Table 4 Logistic regression analysis between alcohol consumption and depression

| Variable | total | event (%) | Non-adjusted model | | Model Ⅰ | | Model Ⅱ | | Model Ⅲ | |
| --- | --- | --- | --- | --- | --- | --- | --- | --- | --- | --- |
|  |  |  | OR (95%CI) | p value | OR (95%CI) | p value | OR (95%CI) | p value | OR (95%CI) | p value |
| Non-drinking | 3436 | 297 (8.6%) | 1(Ref) | | 1(Ref) | | 1(Ref) | | 1(Ref) |  |
| Drinking | 9102 | 731 (8%) | 0.92 (0.8~1.06) | 0.265 | 1.18 (1.01~1.37) | 0.038 | 1.25 (1.07~1.46) | 0.005 | 1.1 (0.93~1.29) | 0.273 |

Model Ⅰ: adjusted gender, age, race, education level, marital status, family income.

Model Ⅱ: adjusted gender, age, race, education level, marital status, family income, smoking, BMI, high intensity exercise, moderate intensity exercise, sleep disorders, hypertension, diabetes, arthritis, coronary disease, thyroid disease, osteoporosis.

Model Ⅲ: adjusted gender, age, race, education level, marital status, family income, smoking, BMI, high intensity exercise, moderate intensity exercise, sleep disorders, hypertension, diabetes, arthritis, coronary disease, thyroid disease, osteoporosis, energy intake, protein intake, carbohydrate intake, total sugars intake, dietary fiber intake, total fat intake, caffeine intake, moisture intake.
